# Antimetastatic activity of a cyclooxygenase-2 inhibitor

**DOI:** 10.1038/sj.bjc.6601967

**Published:** 2004-06-22

**Authors:** G Roche-Nagle, E M Connolly, M Eng, D J Bouchier-Hayes, J H Harmey

**Affiliations:** 1 Department of Surgery, Royal College of Surgeons in Ireland, Education and Research Centre, Beaumont Hospital, Dublin 9, Ireland

**Keywords:** cyclooxygenase-2, angiogenesis, apoptosis, metastasis

## Abstract

Cyclooxygenase-2 (COX-2) expression is increased in breast cancer and surgery has been shown to increase the growth of metastatic tumours. We investigated the effect of selective COX-2 inhibition on the growth of metastases in either an experimental metastasis model or following excision of a murine primary breast tumour. 50 000 4T1 mammary carcinoma cells were injected into the mammary fat pad of female BALB/c mice. When the mean TD reached 8±0.4 mm, tumours were excised and the mice were randomised into two groups (*n*=12 per group) to receive daily intraperitoneal injections of the selective COX-2 inhibitor, SC-236 or drug vehicle for 14 days. Alternatively, experimental metastases were established by tail-vein injection of 50 000 4T1 cells. Mice received either the selective COX-2 inhibitor, SC-236 or drug vehicle for 14 days (*n*=12 per group). SC-236 treatment significantly reduced tumour burden, the number and size of spontaneous metastases following primary tumour excision. SC-236 treatment also reduced tumour burden, the number and size of experimental metastases. Immunohistochemical staining demonstrated that COX-2 inhibition reduced microvessel density and increased apoptosis within both spontaneous and experimental metastases. These data clearly demonstrate that the selective COX-2 inhibitor, SC-236, has potent antimetastatic activity against both spontaneous metastases arising following primary tumour excision and experimental metastases.

In most cancer patients, metastases are already present at time of diagnosis (Fidler and Ellis, 1994) and while surgery remains the mainstay of treatment for primary tumours it may paradoxically enhance growth of residual or metastatic disease (Da Costa *et al*, 1998; Pidgeon *et al*, 1999; Demichelli *et al*, 2001). This may occur as a consequence of an alteration in the balance between pro- and antiangiogenic factors as part of the healing process and removal of the tumour can of itself stimulate tumour growth by removing the source of angiostatin (O'Reilly *et al*, 1997). There is an increase in growth factors in the immediate postoperative period (Nissen *et al*, 1998) and although angiogenesis is essential for wound healing it also plays a key role in the growth and metastasis of tumours (Folkman, 1997). In addition, surgical manipulation may also increase tumour cell dissemination into the bloodstream resulting in the seeding of tumour cells in distant organs and the establishment of metastases (Fisher and Fisher, 1965; Hansen *et al*, 1995). As most cancer patients ultimately die of metastatic disease, it is important to develop therapies that are effective against metastases.

Several studies show that regularly taking aspirin or other conventional nonsteroidal anti-inflammatory drugs (NSAIDs) provides a 40–50% reduction in the relative risk of death by colon cancer, indicating that inhibition of cyclooxygenase (COX), both COX-1 and COX-2, has a chemopreventive effect (Dubois *et al*, 1998). In rodent models of Familial Adenomatous Polyposis (FAP), a genetic disease leading to colon carcinoma, blockade of COX-2, either by gene deletion or pharmacological inhibition of enzyme activity, suppresses intestinal polyp formation (Oshima *et al*, 2001). Cyclooxygenase-2 inhibition also demonstrates chemopreventive activity against colon carcinogenesis in rodent models (Reddy *et al*, 1996). NSAIDS inhibit the activity of both COX enzymes and consequently can inhibit or abolish the effects of prostaglandins (Bjorkman, 1998; Hawkey, 1999). Selective inhibition of the COX-2 isoform has reduced toxicity profile compared to inhibition of both isoforms (Scheiman, 2002). It has been suggested that arachidonic acid, the substrate for COX-2, induces apoptosis and that depletion of arachidonic acid by COX-2 activity decreases apoptosis (Cao *et al*, 2000). We previously demonstrated that COX inhibition reduced the growth of a primary tumour, number and incidence of spontaneous metastases accompanied by increased apoptosis and decreased microvessel density in the primary tumour (Connolly *et al*, 2002).

Prostaglandin E_2_ (PGE_2_)_,_ is produced in large amounts by some tumours and has been implicated in the promotion and growth of malignant tumours (Honn *et al*, 1981). PGE_2_ is produced from arachidonic acid by either of two enzymes: COX-1 is expressed constitutively in most tissues whereas COX-2 is predominantly inducible but these classifications are not absolute. Cyclooxygenase-2 is markedly increased at sites of inflammation and at sites of proliferation such as within tumours (Hawkey, 1999) One of the mechanisms by which PGE_2_ may support tumour growth is by inducing angiogenesis necessary to supply oxygen and nutrients to tumours (Form and Auerbach, 1983). Cyclooxygenase-2 expression has been observed in newly formed blood vessels within tumours, whereas under normal physiological conditions the quiescent vasculature expresses only the constitutive COX-1 enzyme (Masferrer *et al*, 1999).

Growth, invasion and metastasis of many cancers depend on angiogenesis (Folkman, 1990). The current view is that the net balance between endogenous angiogenesis stimulators and inhibitors regulates the ‘switch to the angiogenic phenotype’ (Hanahan and Folkman, 1996). Vascular endothelial growth factor (VEGF)/vascular permeability factor (VPF) is the most potent angiogenic factor identified. VEGF induces both the migration and proliferation of endothelial cells *in vitro* while inhibiting endothelial cell apoptosis (Connolly *et al*, 1989). Therapeutic blockade of VEGF has been shown to inhibit primary and metastatic tumour growth in animal models (Kim *et al*, 1993; Asano *et al*, 1995; Benjamin and Keshet 1997), which has been attributed to an antiangiogenic effect. We and others have recently shown that VEGF can also act as a survival factor for tumour cells, protecting them from apoptosis (Pidgeon *et al*, 2001; Beierle *et al*, 2002; Harmey and Bouchier-Hayes, 2002a).

Although there are many studies showing that COX-2 inhibitors have antitumour activity many of these have used chemically induced tumours and there are few, if any, studies examining antimetastatic effects of COX inhibition. We examined the antimetastatic activity of COX-2 inhibitors in an orthotopic model following excision of the primary tumour and in an experimental metastasis model.

## MATERIALS AND METHODS

### Animals

Female 10–12-week-old BALB/c mice (Charles River Institute, Margate, Kent, UK) were used. The animals were acclimatised for 1 week and caged in groups of five or less in an air-conditioned room at ambient temperature of 21–22°C and 50% humidity under a 12-h light–dark cycle (lights at 0800). Animals were housed in a licensed biomedical facility and all procedures were carried out under animal license guidelines of the Department of Health, Ireland and in accordance with the UK Co-ordinating Committee on Cancer Research (UKCCCR) Guidelines for the Welfare of Animals in Experimental Neoplasia (Workman *et al*, 1998). Animals had *ad libitum* access to animal chow (WM Connolly & Sons Ltd, Kilkenny, Ireland) and water.

### Tumour cells and culture conditions

4T1 tumour cells, a spontaneously metastasising mammary adenocarcinoma cell line were a generous gift from Dr Fred Miller, Duke University. Cells were maintained as monolayer cultures in Dulbecco's modified Eagle medium supplemented with 10% foetal bovine serum (FBS), sodium pyruvate and L-glutamine (Life Technologies, Inc., GIBCO BRL, Paisley, UK). Cell cultures were incubated in an atmosphere of 5% CO_2_ in air at 37°C. Tumour cells were harvested from subconfluent cultures with 0.25% trypsin-0.02% EDTA. Trypsin was neutralised with medium containing 10% FBS, washed three times in phosphate-buffered saline (PBS) and resuspended in PBS at 5 × 10^5^ ml^−1^ for injection. Only single cell suspensions of greater than 90% viability as determined by trypan blue exclusion were used for injections.

### Experimental design

4T1 cells (5 × 10^4^) were injected into the mammary fat pad behind the left forefoot after anaesthesia was induced and maintained with inhalational halothane. Primary tumours were measured on alternate days following injection of tumour cells using a Vernier calipers. Tumour diameter (TD) was calculated as the square root of the product of two perpendicular diameters (Pulaski and Ostrand-Rosenberg, 1998). When mean TD was 8.0±0.4 mm (day 14 postinjection of tumour cells), primary tumours were excised. They were then randomised into one of two groups (*n*=12 per group). The treatment and control groups received daily intraperitoneal injections of either 200 *μ*l of the selective COX-2 inhibitor, SC-236 (6 mg kg^−1^ in 1% v v^−1^ dimethylsulphoxide DMSO) or vehicle (1% DMSO), respectively, for 14 days. Animals were killed and lungs excised. Lungs from seven mice per group were fixed in Bouins solution and the number and size of lung lesions determined with the aid of a dissecting microscope (Yano *et al*, 2000). Lungs from remaining five mice per group were processed for immunohistochemistry.

For studies on experimental metastases BALB/c mice (*n*=12 per group) received 5 × 10^4^ 4T1 cells via tail-vein injection. Controls received daily intraperitoneal injections of 200 *μ*l drug vehicle (1% DMSO) while the treatment group received SC-236 by intraperitoneal injection (6 mg kg^−1^ in 1% DMSO) starting on the day of tumour cell injection. After 14 days, mice were killed, lungs removed, fixed in Bouins solution and the number and size of lung lesions determined as above (*n*=7). Lungs from remaining five mice per group were processed for immunohistochemistry. All experiments were performed in duplicate.

### Serum collection

At the time of killing, animals were anaesthesised with halothane and their chests cleaned with ethanol. Blood was obtained via a closed cardiac puncture by means of a 22-gauge hypodermic needle and a subxiphoid approach. Blood was allowed to clot for 2 h at room temperature and centrifuged for 20 min at 1100 ***g***. Serum was removed, filtered through a 0.22 *μ*m filter and stored at −80°C. VEGF was measured by enzyme-linked immunosorbent assay (ELISA) according to the manufacturer's instructions (R&D Systems, Oxford, UK).

### Quantification of microvessel density

Harvested lungs (*n*=5) were embedded in OCT compound (Miles Inc., Elkhart, IN, USA), snap frozen in liquid nitrogen and stored at −80°C. Microvessel density was determined as described previously by staining with MECA 32 antibody (rat anti-mouse panendothelial antigen (Pharmingen, CA, USA)) (Harmey *et al*, 2002b). For each section, vessels were counted in three high-power fields (× 200 magnification (× 20 objective and × 10 ocular)) as described. Lung sections from each of five mice per group were analysed. Data are expressed as mean±s.e.m.

### Tumour apoptosis

Harvested lungs were embedded in OCT compound (Miles Inc., Elkhart, IN, USA), snap frozen in liquid nitrogen and stored at −80°C. Sections (8 *μ*m) were fixed in cold acetone for 5 min, 1 : 1 acetone : chloroform for 5 min, acetone for 5 min and washed 3 times in phosphate-buffered saline (PBS) for 5 min each. Apoptotic cells within tumour metastases were stained using the *in situ* cell death detection kit according to the manufacturer's instructions (Boehringer Mannheim, East Sussex, UK). Peroxidase activity was visualised by the precipitation of 3,3′-diaminobenzidene (DAB) and sections were lightly counterstained with haematoxylin. Apoptotic cells stained brown against a blue background. Only cells which stained brown and had the morphological appearance of an apoptotic cell were counted as apoptotic cells, necrotic cells were easily distinguished and were excluded. Apoptosis was expressed as the number of apoptotic cells in three high-power fields per section (× 400 magnification (× 40 objective and × 10 ocular)). Lung sections from each of five mice per group were analysed. Data are expressed as mean±s.e.m.

### Statistical analysis

Data are expressed as mean±standard error mean (s.e.m.). Differences between treatment groups were determined by unpaired *t-*test using Instat for Windows statistics package (Graphpad Software Inc). Data were taken as significant where *P*<0.05.

## RESULTS

### Effect of COX-2 inhibition on metastases

We previously demonstrated that COX-2 inhibition decreased the number of metastases from a primary mammary fat pad 4T1 tumour *in situ* (Connolly *et al*, 2002). However, in that study, the observed reduction in metastases could simply have reflected the decreased primary tumour size in mice treated with COX-2 inhibitor rather than a direct effect on the metastases. To clarify the issue, we examined the antimetastatic activity of COX-2 inhibitor following excision of a primary mammary fat pad tumour and in an experimental metastasis model where 4TI tumour cells were injected intravenously.

4T1 mammary adenocarcinoma cells spontaneously metastasise to the lungs from the mammary fat pad (Pulaski and Ostrand-Rosenberg, 1998). Mammary fat pad tumours were excised when they reached a TD of 8±0.4 mm and mice were given SC-236 daily for 14 days after the tumours were excised. SC-236 treatment resulted in a significant reduction in both the number and size of spontaneous lung metastases relative to untreated controls (Table 1

**Table 1:** Effect of selective COX-2 inhibition (SC-236) on metastasis following excision of a 4TI mammary fat pad primary tumour

	**Metastatic burden (lung wt/body wt)**	**Incidence of pulmonary metastasis**	**Number of pulmonary nodules**	**Diameter of nodules (mm)**
	***n*=12**	***n*=12**	***n*=7**	***n*=7**
Control	1.05±0.05	12/12	22.5 (14–57)	0.98 (0.81–1.1)
SC-236	0.86±0.05^a^	12/12	6 (3–34)^b^	0.76 (0.64–0.87)^c^

4T1 cells (5 × 10^4^) were injected into the mammary fat pad of BALB/c female mice and tumour excised when mean diameter was 8±0.4 mm. Following excision daily intraperitoneal injections of vehicle or SC-236 began and continued for 14 days. The animals were killed. The number, size and incidence of pulmonary nodules were recorded. Lung-to-body weight ratio was calculated as a measure of metastatic burden. Data are shown as mean±s.e.m. with nodule data as median (range). Data were analysed by using unpaired *t*-test

a *P*<0.01 *vs* control,

b *P*<0.04 *vs* control,

c *P*<0.002 *vs* control.

). The median number of spontaneous pulmonary metastases was 22.5 (14–57) in the control group, and 6 (3–34) in the SC-236-treated group (*P*=0.04). The lung nodules in the control group were also significantly larger than those in the SC-236-treated mice (median diameter 0.98 (0.81–1.1) mm *vs* 0.76 (0.64–0.87) mm, *P*=0.002). Excised lungs were weighed and metastatic burden expressed as lung-to-body ratio as before (Pidgeon *et al*, 1999). The COX-2-treated mice showed a significant decrease in tumour burden (0.86±0.2) as compared with controls (1.1±0.16) (*P*=0.01).

To confirm the antimetastatic effect of COX-2 inhibition an experimental metastasis model was used. 4T1 tumour cells were injected via lateral tail vein and COX-2 inhibitor was administered daily for 14 days. SC-236 treatment resulted in significantly fewer metastases than in control mice (33 (17–40) *vs* 97 (67–135) (*P*=0.02). Furthermore, the size of metatases in the control group were also significantly larger than those in the SC-236-treated mice (median diameter 0.77 (0.33–0.99) *vs* 0.65 (0.18–0.79) mm, *P*=0.046). (Table 2

**Table 2:** Effect of selective COX-2 inhibition (SC-236) on experimental metastasis

	**Metastatic burden (lung wt/body wt)**	**Incidence of pulmonary metastasis**	**Number of pulmonary nodules**	**Diameter of nodules (mm)**
	***n*=12**	***n*=12**	***n*=7**	***n*=7**
Control	0.9±0.16	12/12	97 (67–135)	0.77 (0.33–0.99)
SC-236	0.66±0.05^a^	12/12	33 (17–40)^b^	0.65 (0.18–0.79)^c^

BALB/c female mice were injected with 5 × 10^4^ 4T1 cells via tail vein followed by daily intraperitoneal injections of vehicle or SC-236 for 14 days. The number, size and incidence of pulmonary nodules were recorded. Lung-to-body weight ratio was calculated as a measure of metastatic burden. Data are shown as mean±s.e.m. with nodule data as median (range). Data were analysed by using unpaired *t*-test,

a *P*<0.02 *vs* control,

b *P*<0.02 *vs* control,

c *P*<0.05 *vs* control.

)

### Effect of COX-2 inhibition on apoptosis within the lung metastases

**Figure 1:**
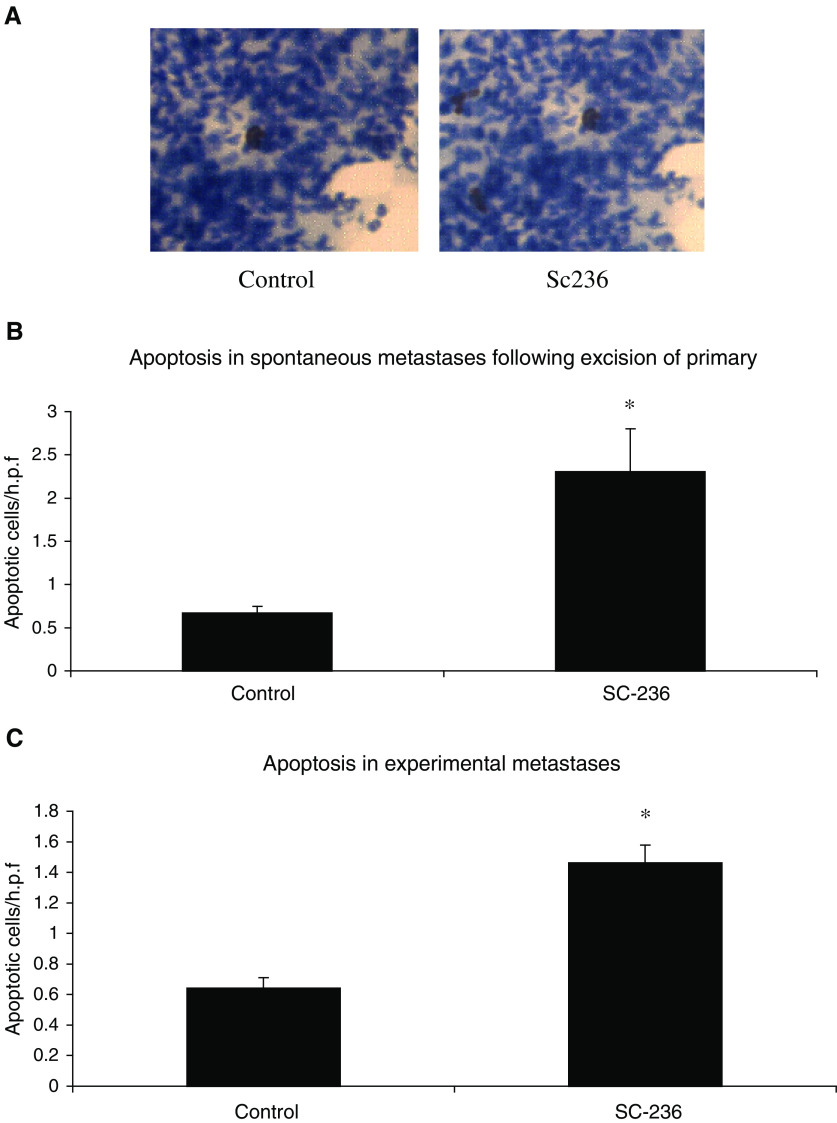
Apoptosis in spontaneous and experimental lung metastases. (**A**) Representative sections showing TUNEL-positive cells. Original magnification × 400. (**B**) Number of TUNEL-positive cells per high-power field (× 400 magnification (× 40 objective and × 10 ocular)) (three high-power fields per section). SC-236 significantly increased apoptosis within spontaneous metastases relative to control mice (one section from each of five mice per group). Data represented as mean±s.e.m. ^*^ *P*=0.006 (*n*=5). (**C**) In experimental metastases SC-236 also significantly increased apoptosis. Data represented as mean±s.e.m. ^*^ *P*=0.006 (*n*=5).

The selective COX-2 inhibitor, SC-236, demonstrated antimetastatic activity against both experimental metastases and spontaneous metastases arising following excision of a primary tumour. The relative levels of apoptosis and proliferation determine net tumour growth (Holmgren *et al*, 1995). In our previous studies, although SC-236 treatment had no effect on proliferation within 4T1 primary tumours, it significantly increased tumour cell apoptosis (Connolly *et al*, 2002). We investigated whether the reduction in number and size of metastases following SC-236 treatment was due to increased apoptosis within the metastases. Apoptotic cells were identified by TUNEL staining and representative sections are shown in Figure 1A

. SC-236 treatment following primary tumour excision resulted in a significant increase in the level of apoptosis within metastases (2.3±0.5 TUNEL-positive cells/h.p.f.) relative to control tumours (0.67±0.08 TUNEL-positive cells/h.p.f.) (*P*=0.006) (Figure 1B). This increased apoptosis was also evident in SC-236-treated mice in the experimental metastasis model (1.46±0.12 TUNEL-positive cells/h.p.f.) relative to untreated controls (0.64±0.07 TUNEL-positive cells/h.p.f.) (*P*=0.006) (Figure 1C).

### COX inhibition reduces tumour angiogenesis

**Figure 2:**
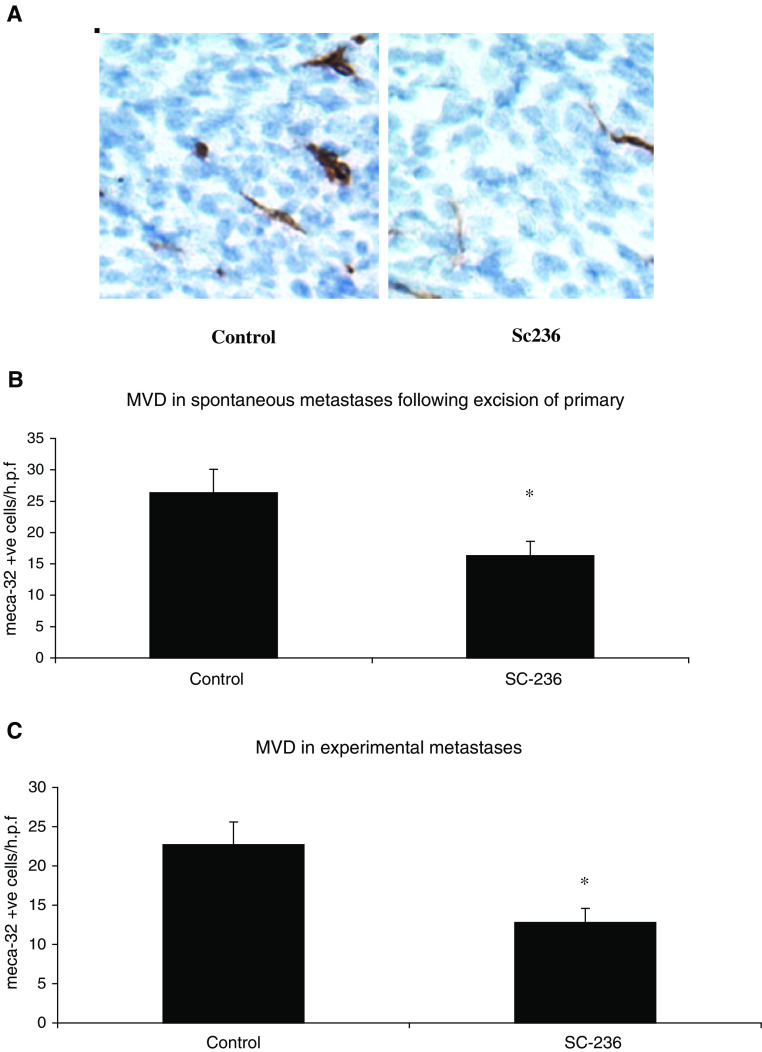
Angiogenesis in spontaneous and experimental lung metastases. (**A**) Representative sections stained for meca-32. Original magnification × 400. (**B**) Microvessel density was assessed by light microscopy following meca-32 staining (one section from each of five mice per group). For each section, vessels were counted in three high-power fields (× 400 magnification (× 40 objective and × 10 ocular)). SC-236 significantly decreased microvessel density within spontaneous metastases relative to control mice. Data are expressed as mean number of vessels/h.p.f.±s.e.m. ^*^P<0.05 *vs* control. (**C**) In experimental metastases SC-236 also significantly decreased microvessel density. Data represented as mean±s.e.m. ^*^P<0.02 (*n*=5).

To further investigate the mechanisms by which the selective COX-2 inhibitor (SC-236) reduced metastasis, we examined the effect of treatment on angiogenesis within the pulmonary metastases following excision of primary tumour. Vascularisation was identified by staining tumours with MECA-32 antibody (Figure 2A

) and the number of vessels/high-power field scored. Treatment with SC-236 significantly reduced angiogenesis in the pulmonary metastases (16±2.2 vessels/h.p.f.), relative to controls (27±4 vessels/h.p.f.) (*P*<0.05) (Figure 2B). The reduction in angiogenesis within metastases was relected in the serum levels of VEGF. Treatment with SC-236 following excision of primary tumour significantly reduced circulating VEGF (30.5±6 pg ml^−1^) relative to controls (83.5±20.9 pg ml^−1^) (*P*=0.02). The antiangiogenic effect of SC-236 treatment was also evident in the experimental metastasis model (12.8±1.8 vessels/h.p.f. in SC-236 treated mice compared to 22.7±2.9 vessels/h.p.f. in controls. (*P*=0.017). Figure 2C. As in the spontaneous metastasis model, treatment with SC-236 significantly reduced circulating VEGF relative to controls (37.6±17.2 *vs* 93.2±55.3 pg ml^−1^) (*P*=0.017).

## DISCUSSION

While surgery results in the successful removal of the primary tumour in the majority of patients, a significant number of patients subsequently die of metastatic disease. The release of proangiogenic factors in the postoperative period may contribute to the growth of previously dormant metastases. In addition, removal of the primary tumour, a source of the endogenous angiogenesis inhibitor, angiostatin, may remove a ‘brake’ on metastatic growth (O'Reilly *et al*, 1997). Previously, we have shown that the surgical insult accelerates the growth of the primary tumour and increases pulmonary metastases (Da Costa *et al*, 1998; Pidgeon *et al*, 1999).

4TI cells originated from a spontaneously occurring BALB/c mouse breast tumour and share many characteristics with human mammary cancers (Pulaski and Ostrand-Rosenberg, 1998). In our previous studies, it was unclear whether decreased metastatic growth in response to COX-2 inhibition was a direct effect on metastases or simply a reflection of decreased primary tumour size (Connolly *et al*, 2002). Here, we used two metastatic models, one where 4T1 cells were injected via tail vein, a model which is limited by the tumour cells arriving in the lungs as part of a first flow phenomenon bypassing the liver. In the second spontaneous metastasis model, a primary tumour was excised. The second is a more clinically relevant orthotopic model of breast cancer, where the primary tumour was excised. Using this orthotopic model of breast cancer excision, we found a highly significant reduction in both the size and number of spontaneous lung metastases in animals receiving daily injections of the selective COX-2 inhibitor, SC-236, postexcision. Furthermore, SC-236 decreased metastatic growth in the experimental metastasis model. These studies clearly demonstrate that the COX-2 inhibitor, SC-236, has a direct antimetastatic effect.

High levels of apoptosis within breast tumours undergoing chemotherapy have been shown to predict a favourable response to treatment (Chang *et al*, 2000). NSAIDS can induce cells to undergo apoptosis *in vitro* (Lu *et al*, 1995). Arachidonic acid stimulates apoptosis, therefore COX-2 inhibition could induce apoptosis by decreasing the conversion of arachidonic acid to prostaglandins (Cao *et al*, 2000). In addition, selective COX-2 inhibition has been shown to induce apoptosis through a cytochrome *C*-dependent pathway in oesophageal cancer cells (Li *et al*, 2001). Other studies have examined the effects of COX inhibitors on behaviour of tumour cells *in vitro*. These data indicate that these same drugs can limit growth of cultured mammary tumour cells, induce cell cycle arrest and increase intracellular ceramide levels (Kundu *et al*, 2001). Clearly, more studies are needed to discern the mechanisms responsible for the antitumour and antimetastatic effects of these drugs. We found that COX-2 inhibition increased tumour cell apoptosis within lung metastases relative to control in both the spontaneous and experimental metastasis models.

Microvessel density within the primary tumour, a measure of the degree of angiogenesis, is an independent predictor of metastatic disease in breast cancer patients (Weidner *et al*, 1991). COX-2 inhibitors have been shown to reduce angiogenesis *in vitro* (Tsujii *et al*, 1998) and *in vivo* (Masferrer 2001). Williams *et al* (2000), found reduced angiogenesis in Lewis lung carcinomas grown in COX-2 knockout (COX-2^−^/^−^) mice when compared to tumours grown in wild-type mice. Here, we show that COX-2 inhibition also decreases angiogenesis within spontaneous and experimental metastases suggesting that in addition to increasing tumour cell apoptosis these drugs exert their antimetastatic effect by also reducing angiogenesis in the metastases. Our hypothesis is further supported by studies indicating a role for COX-2 in the production of angiogenic factors by cancer cells (Tsujii *et al*, 1998), increased cell proliferation (Sheng *et al*, 1988), prevention of apoptosis (Tsujii and DuBois, 1995), increased metastatic potential (Tsujii *et al*, 1997) and the inhibition of immune surveillance (Huang *et al*, 1998).

In addition to inhibiting tumour growth via COX inhibition, it is possible that COX-2 inhibitors may also effect other target molecules. There are studies which show that NSAIDs can have COX-independent activity. First, there is no correlation between sensitivity to NSAID-induced cell death and levels of COX expression in transformed fibroblasts derived from COX-2^−/−^, COX-1^−/−^, or COX-1^−/−^/COX-2^−/−^ mice (Zhang *et al*, 1999). In addition, NSAID derivatives without COX-inhibitory activity, such as sulindac sulphine, can inhibit chemically induced colon carcinogenesis without affecting prostaglandin levels. (Piazza *et al*, 1997). Moreover, high doses of NSAIDs induce apoptosis in colorectal cancer cells with no detectable COX-1 or COX-2 activity (Hanif *et al*, 1996; Williams *et al*, 2000). Several alternative targets have been proposed to explain COX-independent effects of NSAIDs. High doses of ASA (aminosalicylic acid) have been shown to antagonise the NF-*κ*B signalling pathway (Koop and Ghosh, 1994). Peroxisome proliferator-activated receptors (PPARs) are ligand-inducible transcription factors, which belong to the nuclear hormone receptor superfamily. Three different isoforms, PPAR*α*, *δ* and *γ* have been identified in humans, each with a different pattern of expression. Fatty acids and certain fatty acid metabolites have been suggested as endogenous ligands for these receptors. Indomethacin can bind to, and induce transcriptional activity of PPAR*α* and *γ* isoforms, and PPAR*δ* is a target for sulindac (Lehmann *et al*, 1997; He *et al*, 1999). Finally, NSAIDs decrease levels of the antiapoptotic protein, Bcl-X_L_ (Zhang *et al*, 2000). In general, COX-independent effects of NSAIDs require higher concentrations than COX-dependent effects. The concentration of Celecoxib, required to induce apoptosis *in vitro* is in the 50–100 *μ*M range (Williams *et al*, 2000a). This is 10–200-fold higher than serum concentrations required to inhibit tumour growth in mouse models for colorectal and breast cancer (Jacoby *et al*, 2000; Williams *et al*, 2000a). At low concentrations, the best-characterised targets of NSAIDs remain the COX enzymes. (Gupta and Dubois, 2001).

In summary, we have clearly demonstrated that COX-2 inhibitors have a potent antimetastatic effect increasing apoptosis and decreasing angiogenesis within both spontaneous and experimental metastases. The selective COX-2 inhibitors are in everyday clinical use, cheap and with a known side effect-profile. Our data suggest that COX-2 inhibitors may have clinical utility in the management of metastatic disease especially in the perioperative period.

## References

[bib1] Asano M, Yukita A, Matsumoto T, Kondo S, Suzuki H (1995) Inhibition of tumour growth and metastasis by an immunoneutralizing monoclonal antibody to human vascular endothelial growth factor/vascular permeability factor121. Cancer Res 55: 5296–5301 7585591

[bib2] Beierle EA, Strande LF, Chen MK (2002) VEGF upregulates Bcl-2 expression and is associated with decreased apoptosis in neuroblastoma cells. J Pediatr Surg 37: 467–471 11877669 10.1053/jpsu.2002.30868

[bib3] Benjamin LE, Keshet E (1997) Conditional switching of vascular endothelial growth factor (VEGF) expression in tumors: induction of endothelial cell shedding and regression of hemangioblastoma-like vessels by VEGF withdrawal. Proc Natl Acad Sci USA 94: 8761–8766 9238051 10.1073/pnas.94.16.8761 PMC23118

[bib4] Bjorkman DJ (1998) The effect of aspirin and nonsteroidal anti-inflammatory drugs on prostaglandins. Am J Med 105: 8S–12S 9715829 10.1016/s0002-9343(98)00069-2

[bib5] Cao Y, Pearman T, Zimmerman G, McIntyre M, Prescott S (2000) Intracellular unesterified arachidonic acid signals apoptosis. Proc Natl Acad Sci 97: 11280–11285 11005842 10.1073/pnas.200367597 PMC17191

[bib6] Chang J, Ormerod M, Powles TJ, Allred DC, Ashley SE, Dowsett M (2000) Apoptosis and proliferation as predictors of chemotherapy response in patients with breast carcinoma. Cancer 89: 2145–2152 11147583

[bib7] Connolly DT, Heuvelman DM, Nelson R, Olander JV, Eppley BL, Delfino JJ, Siegel NR, Leimgruber RM, Feder J (1989) Tumour vascular permeability factor stimulates endothelial cell growth and angiogenesis. J Clin Invest 84: 1470–1478 2478587 10.1172/JCI114322 PMC304011

[bib8] Connolly EM, Harmey JH, O'Grady T, Foley D, Roche-Nagle G, Kay E, Bouchier-Hayes DJ (2002) Cyclooxygenase inhibition reduces tumour growth and metastasis in an orthotopic model of breast cancer. Br J Cancer 87: 231–237 12107848 10.1038/sj.bjc.6600462 PMC2376100

[bib9] Da Costa ML, Redmond HP, Finnegan N, Flynn M, Bouchier-Hayes D (1998) Laparotomy and laparoscopy differentially accelerate experimental flank tumour growth. Br J Surg 85: 1439–1442 9782033 10.1046/j.1365-2168.1998.00853.x

[bib10] Demichelli RE, Valagussa P, Bonadonna G (2001) Does surgery modify growth kinetics of breast cancer micrometastases. Br J Cancer 85: 490–492 11506484 10.1054/bjoc.2001.1969 PMC2364103

[bib11] Dubois RN, Arbramson SB, Crofford L, Gupta RA, Simon LS, Van De Putte LB, Lipsky PE (1998) Cyclooxygenase in biology and disease. FASEB J 12: 1063–1073 9737710

[bib12] Fidler IJ, Ellis LM (1994) The implications of angiogenesis for the biology and therapy of cancer metastases. Cell 79: 185–188 7525076 10.1016/0092-8674(94)90187-2

[bib13] Fisher ER, Fisher B (1965) Experimental study of factors influencing development of hepatic metastases from circulating tumour cells. Acta Cytol 9: 146–159 14330561

[bib14] Folkman J (1990) What is the evidence that tumours are angiogenesis dependent? J Natl Cancer Inst 82: 4–6 1688381 10.1093/jnci/82.1.4

[bib15] Folkman J (1997) Angiogenesis and angiogenesis inhibition: an overview. EXS 79: 1–8 9002217 10.1007/978-3-0348-9006-9_1

[bib16] Form DM, Auerbach R (1983) PGE2 and angiogenesis. Proc Soc Exp Biol Med 172: 214–218 6572402 10.3181/00379727-172-41548

[bib17] Gupta RA, DuBois RN (2001) Colorectal cancer prevention and treatment by inhibition of cyclooxygenase-2. Nat Rev Cancer 1: 11–21 11900248 10.1038/35094017

[bib18] Hanif R, Pittas A, Feng Y, Koutsos M I, Qiao L, Staiano-Coico L, Shiff SI, Rigas B (1996) Effects of nonsteroidal anti-inflammatory drugs on proliferation and on induction of apoptosis in colon cancer cells by a prostaglandin-independent pathway. Biochem Pharmacol 52: 237–245 8694848 10.1016/0006-2952(96)00181-5

[bib19] Hanahan D, Folkman J (1996) Patterns and emerging mechanisms of the angiogenic switch during tumorigenesis. Cell 86: 353–364 8756718 10.1016/s0092-8674(00)80108-7

[bib20] Hansen E, Wolff N, Knuechel R, Ruschoff J, Hofstaedter F, Taeger K (1995) Tumour cells in blood shed from the surgical field. Arch Surg 130: 387–393 7710337 10.1001/archsurg.1995.01430040049007

[bib21] Harmey JH, Bouchier-Hayes D (2002a) Vascular endothelial growth factor (VEGF), a survival factor for tumour cells: implications for anti-angiogenic therapy. Bioessays 24: 280–283 11891765 10.1002/bies.10043

[bib22] Harmey JH, Bucana CD, Lu W, Byrne AM, McDonnell S, Lynch C, Bouchier-Hayes D, Dong Z (2002b) Lipopolysaccharide-induced metastatic growth is associated with increased angiogenesis, vascular permeability and tumour cell invasion. Int J Cancer 101: 415–422 12216068 10.1002/ijc.10632

[bib23] Hawkey CJ (1999) Cox-2 inhibitors. Lancet 353: 307–314 9929039 10.1016/s0140-6736(98)12154-2

[bib24] Holmgren L, O'Reilly MS, Folkman J (1995) Dormancy of micrometastases: balanced proliferation and apoptosis in the presence of angiogenesis suppression. Nat Med 1: 149–153 7585012 10.1038/nm0295-149

[bib25] He TC, Chan TA, Vogelstein B, Kinzler KW (1999) PPAR delta is an APC-regulated target of nonsteroidal anti-inflammatory drugs. Cell 99: 2443–2447 10.1016/s0092-8674(00)81664-5 PMC3779681 10555149

[bib26] Honn KV, Bockman RS, Mamett LJ (1981) Prostaglandins and cancer: a review of tumor initiation through tumor metastasis. Prostaglandins 21: 833–864 6280245 10.1016/0090-6980(81)90240-9

[bib27] Huang M, Stolina M, Sharma S, Mao JT, Zhu L, Miller PW, Wollman J, Herschman H, Dubinett SM (1998) Non-small cell lung cancer cyclooxygenase-2-dependent regulation of cytokine balance in lymphocytes and macrophages: up-regulation of interleukin 10 and down-regulation of interleukin 12 production. Cancer Res 58: 1208–1216 9515807

[bib28] Jacoby R, Seibert K, Cole CE, Kellof G, Lubet RA (2000) The cyclooxygenase-2 inhibitor celecoxib is a potent preventive and therapeutic agent in the Min mouse model of adenomatous polyposis. Cancer Res 60: 5040–5044 11016626

[bib29] Kim KJ, Li B, Winer J, Armanini M, Gillett N, Phillips HS, Ferrara N (1993) Inhibition of vascular endothelial growth factor-induced angiogenesis suppresses tumour growth *in vivo*. Nature 362: 841–844 7683111 10.1038/362841a0

[bib30] Koop E, Ghosh S (1994) Inhibition of NF-kappa B by sodium salicylate and aspirin. Science 265: 956–959 8052854 10.1126/science.8052854

[bib31] Kundu N, Yang Q, Dorsey R, Fulton AM (2001) Increased cyclooxygenase-2 (Cox-2) expression and activity in a murine model of metastatic breast cancer. Int J Cancer 93: 681–686 11477578 10.1002/ijc.1397

[bib32] Lehmann JM, Lenhard JM, Oliver BB, Ringhold GM, Kliewer SA (1997) Peroxisome proliferator-activated receptor alpha and gamma are activated by indomethacin and other non-steroidal ant-inflammatory drugs. J Biol Chem 272: 3406–3410 9013583 10.1074/jbc.272.6.3406

[bib33] Li M, Wu X, Xu XC (2001) Induction of apoptosis by cyclooxygenase-2 inhibitor NS398 through a cytochrome *C*-dependent pathway in oesophageal cancer cells. Int J Cancer 93: 218–223 11410869 10.1002/ijc.1322

[bib34] Lu X, Xie W, Reed D, Bradshaw WS, Simmons DL (1995) Nonsteroidal anti-inflammatory drugs cause apoptosis and induce cyclooxygenases in chicken embryo fibroblasts. Proc Natl Acad Sci USA 92: 7961–7965 7644521 10.1073/pnas.92.17.7961 PMC41266

[bib35] Masferrer J (2001) Approach to angiogenesis inhibition based on cyclooxygenase-2. Cancer J 7: S144–50 11779086

[bib36] Masferrer JL, Koki AT, Seibert K (1999) Cox-2 inhibitors. A new class of antiangiogenic agents. Ann NY Acad Sci 889: 84–86 10668485 10.1111/j.1749-6632.1999.tb08726.x

[bib37] Nissen NN, Polverini PJ, Koch AE, Volin MV, Gamelli RC, DiPietro CA (1998) Vascular endothelial growth factor mediates angiogenic activity during the proliferative phase of wound healing. Am J Pathol 152: 1445–1452 9626049 PMC1858442

[bib38] O'Reilly MS, Boehm T, Shing Y, Fukai N, Vasios G, Lane WS, Flynn E, Birkhead JR, Olsen BR, Folkman J (1997) Endostatin: an endogenous inhibitor of angiogenesis and tumor growth. Cell 88: 277–285 9008168 10.1016/s0092-8674(00)81848-6

[bib39] Oshima M, Murai N, Kargman S, Arguello M, Luk P, Kwong E, Taketo MM, Evans JF (2001) Chemoprevention of intestinal polyposis in the Apcdelta716 mouse by rofecoxib, a specific cyclooxygenase-2 inhibitor. Cancer Res 61: 1733–1740 11245490

[bib40] Piazza GA, Alberts DS, Hixson LJ, Paranka NS, Li H, Finn T, Bogert C, Guillen JM, Brendal K, Gross PH, Sperl G, Ritchie J, Burt RW, Ellsworth L, Ahen DJ, Pamukcu R (1997) Sulindac sulfine inhibits azoxymethane-induced colon carcinogenesis in rats without reducing prostaglandin levels. Cancer Res 57: 2909–2915 9230200

[bib41] Pidgeon GP, Barr MP, Harmey JH, Foley DA, Bouchier-Hayes DJ (2001) Vascular endothelial growth factor (VEGF) upregulates BCL-2 and inhibits apoptosis in human and murine mammary adenocarcinoma cells. Br J Cancer 85: 273–278 11461089 10.1054/bjoc.2001.1876 PMC2364032

[bib42] Pidgeon GP, Harmey JH, Kay E, Da Costa M, Redmond HP, Bouchier-Hayes DJ (1999) The role of endotoxin/lipopolysaccharide in surgically induced tumour growth in a murine model of metastatic disease. Br J Cancer 81: 1311–1317 10604727 10.1038/sj.bjc.6694369 PMC2362969

[bib43] Pulaski BA, Ostrand-Rosenberg S (1998) Reduction of established spontaneous mammary carcinoma metastases following immunotherapy with major histocompatibility complex class II and B7.1. Cancer Res 38: 1486–1493 9537252

[bib44] Reddy BS, Rao CV, Seibert K (1996) Evaluation of cyclooxygenase-2 inhibitor for potential chemopreventive properties in colon carcinogenesis. Cancer Res 56: 4566–4569 8840961

[bib45] Scheiman JM (2002) Outcome studies of the gastrointestinal safety of cyclooxygenase-2 inhibitors. Cleve Clin J Med 69: SI40–SI46 12086292 10.3949/ccjm.69.suppl_1.si40

[bib46] Sheng H, Williams CS, Shao J, Liang P, DuBois RN (1988) Induction of cyclooxygenase-2 by activated Ha-ras oncogene in Rat-1 fibroblasts and the role of mitogen-activated protein kinase pathway. J Biol Chem 273: 22120–22127 10.1074/jbc.273.34.22120 9705357

[bib47] Tsujii M, DuBois RN (1995) Alterations in cellular adhesion and apoptosis in epithelial cells overexpressing prostaglandin endoperoxide synthase 2. Cell 83: 493–501 8521479 10.1016/0092-8674(95)90127-2

[bib48] Tsujii M, Kawano S, DuBois RN (1997) Cyclooxygenase-2 expression in human colon cancer cells increases metastatic potential. Proc Natl Acad Sci USA 94: 3336–3340 9096394 10.1073/pnas.94.7.3336 PMC20370

[bib49] Tsujii M, Kawano S, Tsujii S, Sawaoka H, Hori M, Du Bois RN (1998) Cyclooxygenase regulates angiogenesis induced by colon cancer cells. Cell 93: 705–716 9630216 10.1016/s0092-8674(00)81433-6

[bib50] Weidner MD, Semple JP, Welch WR, Folkman J (1991) Tumour angiogenesis and metastasis - correlation in invasive breast carcinoma. N Engl J Med 324: 1–8 10.1056/NEJM199101033240101 1701519

[bib51] Williams CS, Tsujii M, Reese J, Dey SK, DuBois RN (2000) Host cyclooxygenase-2 modulates carcinoma growth. J Clin Invest 105: 1589–1594 10841517 10.1172/JCI9621 PMC300858

[bib52] Williams CS, Watson AJ, Sheng H, Helou R, Shao J, DuBois RN (2000a) Celecoxib prevents tumour growth *in vivo* without toxicity to normal gut: lack of correlation between *in vitro* and *in vivo* models. Cancer Res 60: 6045–6051 11085526

[bib53] Workman P, Twentyman P, Balkwill F, Balmain A, Chaplin D, Double J, Embleton J, Newell D, Raymond R, Stables J, Stephens T, Wallace J (1998) United Kingdom Co-ordinating Committee on Cancer Research (UKCCCR) Guidelines for the Welfare of Animals in Experimental Neoplasia (Second Edition). Br J Cancer 77: 1–10 10.1038/bjc.1998.1 PMC2151254 9459138

[bib54] Yano S, Herbst RS, Shinohara H, Knighton B, Bucana CD, Killion JJ, Wood J, Fidler IJ (2000) Treatment for malignant pleural effusion of human lung adenocarcinoma by inhibition of vascular endothelial growth factor receptor tyrosine kinase phosphorylation. Clin Cancer Res 6: 957–965 10741721

[bib55] Zhang L, Yu J, Park BH, Kinzler KW, Vogelstein B (2000) Role of BAX in the apoptotic response to anti-cancer agents. Science 290: 989–992 11062132 10.1126/science.290.5493.989

[bib56] Zhang X, Morham SG, Langenbach R, Young DA (1999) Malignant transformation and antineoplastic actions of non steroidal anti-inflammatory drugs (NSAIDs) on cyclooxygenase-null embryo fibroblasts. J Exp Med 190: 451–459 10449516 10.1084/jem.190.4.451 PMC2195603

